# Validation list no. 231: valid publication of new names and new combinations effectively published outside the IJSEM

**DOI:** 10.1099/ijsem.0.007238

**Published:** 2026-10-01

**Authors:** Aharon Oren, Markus Göker

**Affiliations:** 1 The Institute of Life Sciences, The Hebrew University of Jerusalem, The Edmond J. Safra Campus, 9190401 Jerusalem, Israel; 2 Leibniz Institute DSMZ – German Collection of Microorganisms and Cell Cultures, Inhoffenstrasse 7B, 38124 Braunschweig, Germany

The purpose of this list is to publish in the International Journal of Systematic and Evolutionary Microbiology (IJSEM) those new names and new combinations that were effectively published outside the IJSEM, as set forth in Rule 27 of the International Code of Nomenclature of Prokaryotes (2025 Revision) [1]. Authors and other individuals wishing to have new names and/or combinations included in future lists should send an electronic copy of the published paper to the IJSEM Editorial Office for confirmation that all other requirements for valid publication have been met. It is also a requirement of the IJSEM and the International Code of Nomenclature of Prokaryotes (ICNP) that authors of new species, new subspecies and new combinations provide evidence that the type strains are deposited in two publicly accessible culture collections in two different countries. It should be noted that the date of valid publication of these new names and combinations is the date of publication of this list, not the date of the original publication of the names and combinations. The authors of the new names and combinations are as given below.

Inclusion of a name on these lists validates the publication of the name and thereby makes it available in the nomenclature of prokaryotes. Inclusion of a name on these lists is part of the requirements for the valid publication of an effectively published name. Indeed, some of these names may, in time, be considered later synonyms or it may be proposed to transfer the organisms to another genus, thus necessitating the creation of a new combination. Conversely, it may be proposed to transfer an organism back to an earlier genus and to regard the basonym or an earlier new combination as the correct name instead of a more recent new combination. The ICNP does not decide on such taxonomic opinions.

**Table:** 

Name/author	Proposed as	Nomenclatural type^1^	Priority^2^	Reference
*Achromobacter colocynthidis* corrig. Ait Si Mhand *et al*. 2026, 12^3,4^	sp. nov.	AGC45 (=CCMM B1338=ITEM 20048)	44	[2]
*Achromobacter maghribensis* Ait Si Mhand *et al*. 2026, 13^3,5^	sp. nov.	AGC69 (=CCMM B1340=ITEM 20050)	44	[2]
*Achromobacter semiaridus* corrig. Ait Si Mhand *et al*. 2026, 13^3,6^	sp. nov.	AGC39 (=CCMM B1336=ITEM 20047)	44	[2]
*Acidaminococcus faecalis* Jang *et al*. 2026, 9^3^	sp. nov.	DS4831 (=JCM 37388=KCTC 25843)	43	[3]
*Acidithiobacillus sibiricus* Panyushkina *et al*. 2026, 13^3^	sp. nov.	A1 (=CGMCC 1.62200=KCTC 8561)	33	[4]
*Acinetobacter endophyllus* corrig. Mouhib *et al*. 2025, 13^3,7^	sp. nov.	AGC35 (=CCMM B1335=ITEM 20056)	80	[5]
*Acinetobacter pullicarnis* Han *et al*. 2020, 731^8^	sp. nov.	S23 (=JCM 33150=KACC 19921)	92	[6]
*Actinotignum cystesis* Winterflood *et al*. 2026, 8^3,9^	sp. nov.	usb_actsch_34 (=CCUG 77579=LMG 33441)	22	[7]
*Actinotignum inguinis* Winterflood *et al*. 2026, 8^3^	sp. nov.	usb_actsch_31 (=CCUG 77578=LMG 33440)	22	[7]
*Actinotignum lotii* Winterflood *et al*. 2026, 8^3^	sp. nov.	usb_actsch_203 (=CCUG 77583=LMG 33445)	22	[7]
*Actinotignum saccati* Winterflood *et al*. 2026, 9^3^	sp. nov.	usb_actsch_70 (=CCUG 77581=LMG 33443)	22	[7]
*Actinotignum stranguriae* Winterflood *et al*. 2026, 9^3^	sp. nov.	usb_actsch_61 (=CCUG 77580=LMG 33442)	22	[7]
*Actinotignum urematis* Winterflood *et al*. 2026, 9^3,10^	sp. nov.	usb_actsch_93 (=CCUG 77582=LMG 33444)	22	[7]
*Actinotignum vesiculae* Winterflood *et al*. 2026, 9^3^	sp. nov.	usb_actsch_30 (=CCUG 77577=LMG 33439)	22	[7]
*Allocellulophaga* Nakajima *et al*. 2026, 10^3^	gen. nov.	*Allocellulophaga tsushimaensis*	50	[8]
*Allocellulophaga tsushimaensis* Nakajima *et al*. 2026, 12^3^	sp. nov.	TSM-5 (=KCTC 52508=NBRC 112430)	50	[8]
*Aquidulcibacteraceae* Hallgren *et al*. 2026, 1^3^	fam. nov.	*Aquidulcibacter*	27	[9]
*Bacillus crepusculi* Williams *et al*. 2026, 14^3,11^	sp. nov.	28A-2 (=DSM 111873=NCIMB 15401)	2	[10]
*Bacillus ferrooxidans* Zhou *et al*. 2018, 476^12^	sp. nov.	YT-3 (=CCTCC AB 2017049=KCTC 33875)	8	[11]
*Bifidobacterium intestinale* Shamsuzzaman *et al*. 2026, 9^3^	sp. nov.	M3-R-103 (=CGMCC 1.19415=KCTC 25443)	70	[12]
*Billgrantia hypersalina* Moran-Olvera *et al*. 2025, 12^3,13^	sp. nov.	LNSP4103-1 (=ATCC TSD-413=CAIM 1962)^14^	39	[13]
*Blautia brookingsensis* corrig. Ghimire *et al*. 2020, 5^3,15^	sp. nov.	SG772 (=CCOS 1888=DSM 107275)	52	[14]
*Blautia caeci* Shamsuzzaman et al. 2026, 9^3^	sp. nov.	C3-R-101 (=CGMCC 1.17981=KCTC 25440)	70	[12]
*Bordetella saccharivorans* Akita *et al*. 2026, 6^3^	sp. nov.	NSA17 (=HUT-8147=KCTC 82065)	31	[15]
*Brakhagea* Kumar *et al*. 2026, 8^3^	gen. nov.	*Brakhagea baltica*	23	[16]
*Brakhagea aquatica* Kumar *et al*. 2026, 10^3^	sp. nov.	SH501 (=CECT 30906=KCTC 102088)	23	[16]
*Brakhagea baltica* Kumar *et al*. 2026, 8^3^	sp. nov.	SH449 (=DSM 116586=KCTC 102025)	23	[16]
*Brakhagea lacunae* Kumar *et al*. 2026, 9^3^	sp. nov.	SH551 (=CECT 30846=DSM 116785=KCTC 102023)	23	[16]
*Brakhagea slesvicensis* Kumar *et al*. 2026, 9^3^	sp. nov.	SH467 (=DSM 116378=KCTC 102026)	23	[16]
*Cohnella capsici* Wang *et al*. 2015, 137	sp. nov.	YN-59 (=CGMCC 1.12046=JCM 19168)	83	[17]
*Corynebacterium lipophilum* Luo *et al*. 2023, 1098	sp. nov.	MC-18 (=CCTCCAB 2020201=NBRC 115144)	87	[18]
*Cupriavidus smyrnaeus* Sahin and Tani 2026, 7^3^	sp. nov.	TA19 (=CCM 7673=DSM 28348=NBRC 107719)	29	[19]
*Denitratimonas organivorans* An *et al*. 2026, 11^3^	sp. nov.	CY0512 (=GDMCC 1.3762=JCM 36229)	11	[20]
*Denitrificimonas halotolerans* Wang *et al*. 2024, 9^3,16^	sp. nov.	JX-1 (=KCTC 8395=MCCC 1K08958)	103	[21]
*Desulforudaceae* Karnachuk *et al*. 2026, 8^3^	fam. nov.	*Desulforudis*	14	[22]
*Desulforudis* (*ex* Chivian *et al*. 2008) Karnachuk *et al*. 2026, 7^3^	gen. nov.	*Desulforudis audaxviator*	14	[22]
*Desulforudis audaxviator* (*ex* Chivian *et al*. 2008) Karnachuk *et al*. 2026, 8^3^	sp. nov. [pro-basonym: *Desulforudis audaxviator* Chivian *et al*. 2008]	BYF (=JCM 39285=VKM B-3564)^17^	14	[22]
*Desulfosarcina ovata* subsp. *ovata* (Kuever *et al*. 2006) Watanabe *et al*. 2020, 4^3^	comb. nov. [basonym: *Desulfosarcina ovata* Kuever *et al*. 2006]	oXyS1 (=DSM 13228=JCM 12297)^18^	72	[23]
*Desulfosarcina ovata* subsp. *sediminis* Watanabe *et al*. 2020, 4^3^	subsp. nov.	28bB2T (=DSM 23484=NBRC 106234)^19^	72	[23]
*Desulfovibrio mangrovi* Zhou *et al*. 2023, 508	sp. nov.	FT415 (=GDMCC 1.3410=KCTC 25525)	60	[24]
*Devosia litorisediminis* Park *et al*. 2022, 4^3^	sp. nov.	BSSL-BM10 (=KACC 21633=NBRC 115152)	90	[25]
*Draconibacterium aestuarii* Wang *et al*. 2024, 6^3^	sp. nov.	Z1-6 (=KCTC 92310=MCCC 1K07533)	59	[26]
*Erythrobacter mangrovi* Ye *et al*. 2020, 1433	sp. nov.	EB310 (=KCTC 72109=MCCC 1K03690)	65	[27]
*Flavobacterium difficile* Chen *et al*. 2021, 4457	sp. nov.	KDG-16 (=BCRC 81194=LMG 31332)	100	[28]
*Flavobacterium maulense* Avendaño-Herrera *et al*. 2026, 10^3^	sp. nov.	T-16 (=CECT 30411=RGM 3224)	42	[29]
*Gelidimonas* Grzesiak *et al*. 2026, 10^3^	gen. nov.	*Gelidimonas denitrificans*	81	[30]
*Gelidimonas denitrificans* Grzesiak *et al*. 2026, 10^3^	sp. nov.	D11 (=DSM 120166=LMG 34060)	81	[30]
*Gelidimonas diazotrophica* Grzesiak *et al*. 2026, 10^3^	sp. nov.	D2 (=DSM 120165=LMG 34059)	81	[30]
*Gephyromycinifex* Zhu *et al*. 2021, 2014	gen. nov.	*Gephyromycinifex aptenodytis*	96	[31]
*Gephyromycinifex aptenodytis* Zhu *et al*. 2021, 2014	sp. nov.	NJES-13 (=CCTCC 2019007=KCTC 49281)	96	[31]
*Gracilibacillus eburneus* Guan *et al*. 2018, 427	sp. nov.	YIM 93565 (=CCTCCAB 2013249=DSM 23710)	105	[32]
*Jonesia luteola* Li *et al*. 2015, 617	sp. nov.	YIM 93067 (=CCTCCAB 2014350=DSM 21367)	94	[33]
*Haloarchaeobius caseinilyticus* Xu *et al*. 2026, 9^3^	sp. nov.	DFWS5 (=CGMCC 1.62757=JCM 37971=KCTC 4384=MCCC 4K00270)	25	[34]
*Haloarchaeobius lipolyticus* Xu *et al*. 2026, 10^3^	sp. nov.	TZWWS8 (=CGMCC 1.63137=KCTC 4405=MCCC 4K00311)	25	[34]
*Haloarchaeobius marinus* Xu *et al*. 2026, 10^3^	sp. nov.	HRN-SO-5 (=CGMCC 1.62838=KCTC 4396=MCCC 4K00286)	25	[34]
*Haloarchaeobius ordinarius* Xu *et al*. 2026, 9^3^	sp. nov.	DT45 (=CGMCC 1.63105=KCTC 4386=MCCC 4K00268	25	[34]
*Haloarchaeobius pelagicus* Xu *et al*. 2026, 10^3^	sp. nov.	DYHT-AS-18 (=CGMCC 1.62836=JCM 37972=KCTC 4403=MCCC 4K00284)	25	[34]
*Halocola* Hu *et al*. 2026, 19^3,20^	gen. nov.	*Halocola ammonii*	19	[35]
*Halocola ammonii* Hu *et al*. 2026, 19^3,21^	sp. nov.	DA487 (=KCTC 102213=MCCC 1H01450)	19	[35]
*Halocolaceae* Hu *et al*. 2026, 20^3^	fam. nov.	*Halocola*	19	[35]
*Hanstruepera marina* Ding *et al*. 2022, 10^3^	sp. nov.	NBU2968 (=KCTC 82913=MCCC 1K06392)	66	[36]
*Hydrosulfovibrio* Zhang *et al*. 2025, 8^3,22^	gen. nov.	*Hydrosulfovibrio ferrireducens* ^23^	108	[37]
*Hydrosulfovibrio ferrireducens* Zhang *et al*. 2025, 8^3,24^	sp. nov.	CB1MN (=DSM 117064=MCCC 1K08991)	108	[37]
*Jannaschia pagri* Kuwata *et al*. 2024, 7^3^	sp. nov.	AI_62 (=DSM 115720=NBRC 115938)	47	[38]
*Janthinobacterium saanense* Kumar *et al*. 2026, 12^3,25^	sp. nov.	S3T4 (=DSM 120996=HAMBI 3823)^26^	20	[39]
*Janthinobacterium silvisoli* Kumar *et al*. 2026, 12^3,25^	sp. nov.	K2Li3 (=DSM 121001=HAMBI 3838)^26^	20	[39]
*Jindonia* Park *et al*. 2017, 425	gen. nov.	*Jindonia aestuariivivens*	62	[40]
*Jindonia aestuariivivens* Park *et al*. 2017, 425	sp. nov.	JDTF-65 (=KCTC 52564=NBRC 112534)	62	[40]
*Kamagatella* Kondo and Kataoka 2026, 11^3^	gen. nov.	*Kamagatella elastica*	13	[41]
*Kamagatella elastica* Kondo and Kataoka 2026, 11^3^	sp. nov.	SANA (=DSM 120061=JCM 39611)	13	[41]
*Kamagatellaceae* Kondo and Kataoka 2026, 11^3^	fam. nov.	*Kamagatella*	13	[41]
*Kineococcus arenarius* Huang *et al*. 2026, 382	sp. nov.	SYSU DK007 (=CGMCC 1.61926=KCTC 59337=MCCC 1K09197)^27^	32	[42]
*Kineococcus arenarius* subsp. *arenarius* (Huang *et al*. 2026) Huang *et al*. 2026, 382	comb. nov.	SYSU DK007 (=CCTCCAA 2024017=CGMCC 1.61926=KCTC 59337=MCCC 1K09197)^27^	32	[42]
*Kineococcus arenarius* subsp. *brunneus* Huang *et al*. 2026, 382	subsp. nov.	SYSU DK019 (=KCTC 59349=MCCC 1K09209)^28^	32	[42]
*Kineococcus aridisoli* Huang *et al*. 2026, 382	sp. nov.	SYSU DK006 (=CGMCC 1.61925=KCTC 59336=MCCC 1K09196)^29^	32	[42]
*Kineococcus auxinigenes* Huang *et al*. 2026, 383	sp. nov.	SYSU DK024 (=CCTCCAA 2024025=CGMCC 1.61941=KCTC 59354=MCCC 1K09321)	32	[42]
*Kineococcus deserti* Huang *et al*. 2026, 381	sp. nov.	SYSU DK003 (=KCTC 59333=MCCC 1K09194)	32	[42]
*Kineococcus esterisolvens* Huang *et al*. 2026, 383	sp. nov.	SYSU DK018 (=CCTCCAA 2024020=CGMCC 1.61937=KCTC 59348=MCCC 1K09208	32	[42]
*Kineococcus gurbantunggutensis* Huang *et al*. 2026, 382	sp. nov.	SYSU DK005 (=CGMCC 1.61924=KCTC 59335=MCCC 1K09195)^30^	32	[42]
*Kineococcus haloalkalitolerans* Huang *et al*. 2026, 381	sp. nov.	SYSU DK004 (=CCTCCAA 2024014=KCTC 59334=MCCC 1K09299)	32	[42]
*Kineococcus lubricus* Huang *et al*. 2026, 381	sp. nov.	SYSU DK001 (=CCTCCAA 2024011=CGMCC 1.61922=KCTC 59331)^31^	32	[42]
*Kineococcus rugosus* Huang *et al*. 2026, 381	sp. nov.	SYSU DK002 (=CCTCCAA 2024012=KCTC 59332=MCCC 1K09193)^32^	32	[42]
*Kistimonas ophiarachnellae* Kawano *et al*. 2026, 8^3^	sp. nov.	ToK13 (=JCM 36222=KCTC 8064)	17	[43]
*Kothea* Kumar *et al*. 2026, 7^3^	gen. nov.	*Kothea flammea*	110	[44]
*Kothea flammea* Kumar *et al*. 2026, 7^3^	sp. nov.	SH139 (=DSM 116129=KCTC 102020)	110	[44]
*Kushneria halocnemicola* Romano-Rodríguez *et al*. 2026, 10^3^	sp. nov.	TE3 (=CECT 31276=LMG 34081)	54	[45]
*Labrenzia callyspongiae* Park *et al*. 2019, 1973	sp. nov.	CE80 (=JCM 31309=KCTC 42849)	74	[46]
*Lacticaseibacillus boliensis* Wang *et al*. 2026, 4^3^	sp. nov.	GG6-2 (=GDMCC 1.6008=JCM 38218)	12	[47]
*Lacunimicrobium* Kabuu *et al*. 2025, 7^3^	gen. nov.	*Lacunimicrobium album*	109	[48]
*Lacunimicrobium album* Kabuu *et al*. 2025, 7^3^	sp. nov.	SH248 (=CECT 30703=JMRC:STH:01007)	109	[48]
*Listeria londonensis* corrig. (Boerlin *et al*. 1992) Bouznada *et al*. 2025, 19^3,33^	comb. nov. [basonym: *Listeria ivanovii* subsp. *londoniensis* Boerlin *et al*. 1992]^34^	CNL 89/5081 (=CCUG 36672=DSM 12491)^35^	98	[49]
*Marinovum faecis* Yoon *et al*. 2017, 967	sp. nov.	YP194 (=KCCM 90263=NBRC 111905)	45	[50]
*Massilibacterium* Tidjani Alou *et al*. 2016, 106^36^	gen. nov.	*Massilibacterium senegalense* ^37^	46	[51]
*Massilibacterium senegalense* Tidjani Alou *et al*. 2016, 108^38^	sp. nov.	mt8 (=CSUR P1510=DSM 100455)	46	[51]
*Massilibacteroides* corrig. Jneid *et al*. 2018, 7^39^	gen. nov.	*Massilibacteroides vaginae*	88	[52]
*Massilibacteroides vaginae* corrig. Jneid *et al*. 2018, 7	sp. nov.	MV12 (=CSUR P1477=DSM 100704)	88	[52]
*Methanoculleus bovis* Trubitsyn *et al*. 2025, 909^40^	sp. nov.	7T (=JCM 39547=VKM B-3409)^41^	15	[53]
*Microbacterium horti* Akter *et al*. 2016, 238^42^	sp. nov.	THG-SL1 (=CCTCCAB 2015117=KACC 18286)	7	[54]
*Micromonospora aridisoli* Han *et al*. 2025, 1128	sp. nov.	SYSU D00755 (=KCTC 49600=MCCC 1K06224)	35	[55]
*Micromonospora desertarenae* Han *et al*. 2025, 1128	sp. nov.	SYSU D00602 (=KCTC 49602=MCCC 1K06192)	35	[55]
*Micromonospora marincola* Han *et al*. 2025, 1127	sp. nov.	SYSU B006 (=KCTC 49613=MCCC 1K06235)	35	[55]
*Micromonospora xinjiangensis* Han *et al*. 2025, 1129	sp. nov.	SYSU D00963 (=KCTC 49606=MCCC 1K06226)	35	[55]
*Mycobacterium cajalii* Tristancho-Baró *et al*. 2026, 11^3^	sp. nov.	HUMS_1102779-3 (= CECT 31296=DSM 120455)^26^	30	[56]
*Mycobacterium ostraviense* Jagielski and Ulmann 2020, 24^3^	sp. nov.	241/15 (=DSM 110538=ITM-501146)	76	[57]
*Mycolicibacterium arenosum* Jeong *et al*. 2024, 7^3^	sp. nov.	CAU 1645 (=KCTC 49724=MCCC 1K07087)	84	[58]
*Nasibacterium* Li *et al*. 2026, 332	gen. nov.	*Nasibacterium caprae*	41	[59]
*Nasibacterium caprae* Li *et al*. 2026, 332	sp. nov.	ZY210820 (=CCTCCAB 2021475=NBRC 115474)	41	[59]
*Nibribacter ruber* Park *et al*. 2020, 2184	sp. nov.	BT10 (=KCTC 62607=NBRC 114383)	18	[60]
*Nisaea sediminum* Zhu *et al*. 2021, 2119	sp. nov.	NBU1469 (=KCTC 82224=MCCC 1K04763)	68	[61]
*Nitratireductor arenosus* Baek *et al*. 2020, 5^3^	sp. nov.	CAU 1489 (=KCTC 62997=NBRC 113694)^43^	71	[62]
*Nitratireductor luteus* Yang *et al*. 2023, 227^44^	sp. nov.	SC-ST17 (=KCTC 92119=MCCC 1K07309)^45^	107	[63]
*Njordella* Staack *et al*. 2026, 7^3^	gen. nov.	*Njordella aestuarii*	111	[64]
*Njordella aestuarii* Staack *et al*. 2026, 8^3^	sp. nov.	SH451 (=CECT 30907=KCTC 102089)	111	[64]
*Njordella calcicola* Staack *et al*. 2026, 9^3^	sp. nov.	SH302 (=DSM 116728=KCTC 102091)	111	[64]
*Nocardia anocheti* Somphong *et al*. 2026, 8^3^	sp. nov.	AG03 (=NBRC 115864=TBRC 16206)	24	[65]
*Nocardioides aquigermanicae* corrig. Zhou *et al*. 2026, 9^3,46^	sp. nov.	WG_orange (=DSM 117947=KCTC 59414)	4	[66]
*Nonomuraea corallina* Ngamcharungchit *et al*. 2023, 7^3,47^	sp. nov.	MCN248 (=NBRC 115966=TBRC 17110)	49	[67]
*Oceanicaulis satelles* (Kevbrin *et al*. 2021) Hallgren *et al*. 2026, 2^3^	comb. nov. [basonym: *Alkalicaulis satelles* Kevbrin *et al*. 2021]	G192 (=KCTC 72746=VKM B-3306)	27	[9]
*Paenibacillus bryophylli* corrig. Liu *et al*. 2018, 2272^48^	sp. nov.	L201 (=GDMCC 1.1251=KCTC 33951)	77	[68]
*Paenibacillus rubeinfantis* corrig. Pham *et al*. 2017, 11^49^	sp. nov.	MT18 (=CSUR P2076=DSM 101191)	9	[69]
*Parabacteroides vesiculifaciens* Chaplin *et al*. 2026, 18^3^	sp. nov.	ASD2025 (=JCM 37967=VKM B-3926)	16	[70]
*Paraburkholderia dokdonensis* Madhaiyan *et al*. 2022, 4^3^	sp. nov.	DCR-13 (=LMG 30889=KCTC 62811)	61	[71]
*Paracellulophaga* Nakajima *et al*. 2026, 2^3^	gen. nov.	*Paracellulophaga baltica*	51	[72]
*Paracellulophaga algicola* (Bowman 2000) Nakajima *et al*. 2026, 2^3^	comb. nov. [basonym: *Cellulophaga algicola* Bowman 2000]	IC166 (=CIP 107446=DSM 14237)^50^	51	[72]
*Paracellulophaga baltica* (Johansen *et al*. 1999) Nakajima *et al*. 2026, 2^3^	comb. nov. [basonym: *Cellulophaga baltica* Johansen *et al*. 1999]	NN015840 (=ATCC 700862=DSM 24729)	51	[72]
*Paracellulophaga pacifica* (Nedashkovskaya *et al*. 2004) Nakajima *et al*. 2026, 2^3^	comb. nov. [basonym: *Cellulophaga pacifica* Nedashkovskaya *et al*. 2004]	KMM 3664 (=JCM 11735=LMG 21938)	51	[72]
*Paracellulophaga tyrosinoxydans* (Kahng *et al*. 2009) Nakajima *et al*. 2026, 2^3^	comb. nov. [basonym: *Cellulophaga tyrosinoxydans* Kahng *et al*. 2009]	EM41 (=DSM 21164=KCTC 22297)	51	[72]
*Parahaemophilus* Segui *et al*. 2026, 14^3^	gen. nov.	*Parahaemophilus parainfluenzae* ^51^	6	[73]
*Parahaemophilus parainfluenzae* (Rivers 1922) Segui *et al*. 2026, 14^3^	comb. nov. [basonym: *Haemophilus parainfluenzae* Rivers 1922 (Approved Lists 1980)]^34^	ATCC 33392 (=CCUG 12836=DSM 8978)^52^	6	[73]
*Parahaemophilus pittmaniae* (Nørskov-Lauritsen *et al*. 2005) Segui *et al*. 2026, 15^3^	comb. nov. [basonym: *Haemophilus pittmaniae* Nørskov-Lauritsen *et al*. 2005]^34^	HK 85 (=CCUG 48703=NCTC 13334)^53^	6	[73]
*Parasediminibacterium schistidii* Wong *et al*. 2026, 9^3^	sp. nov.	NIPR152 (=JCM 36343=KCTC 102165)	37	[74]
*Pasteurella felis* (Inzana *et al*. 1999) Segui *et al*. 2026, 17^3^	comb. nov. [basonym: *Haemophilus felis* Inzana *et al*. 1999]^34^	TI189 (=CCUG 31170=DSM 21192)^54^	6	[73]
*Pedobacter panacis* Singh *et al*. 2017, 241	sp. nov.	DCY108 (=CCTCCAB 2015196=KCTC 42748)	99	[75]
*Pedobacter puniceus* corrig. Yang *et al*. 2020, 4190^55^	sp. nov.	HX-22-1 (=CCTCCAB 2019348=KCTC 72655)	93	[76]
*Pilosibacter rotomagensis* Gloanec *et al*. 2026, 10^3^	sp. nov.	HC1M1C21 (=DSM 119410=LMG 33828)	28	[77]
*Poindextera* Hallgren *et al*. 2026, 1^3^	gen. nov.	*Poindextera montana*	27	[9]
*Poindextera montana* (Tang *et al*. 2024) Hallgren *et al*. 2026, 1^3^	comb. nov. [basonym: *Phenylobacterium montanum* Tang *et al*. 2024]	S6 (=GCMCC 1.18594=NBRC 115419)	27	[9]
*Prevotella pectinovora* Nograšek *et al*. 2015, 508^56^	sp. nov.	P4-76 (=DSM 29996=ZIM B1020)	64	[78]
*Pseudactinobacillus* Segui *et al*. 2026, 16^3^	gen. nov.	*Pseudactinobacillus ducreyi* ^51^	6	[73]
*Pseudactinobacillus ducreyi* (Neveu-Lemaire 1921) Segui *et al*. 2026, 17^3^	comb. nov. [basonym: *‘Coccobacillus ducreyi*’ Neveu-Lemaire 1921]	ATCC 33940 (=CCUG 4438=NCTC 10945)^57^	6	[73]
*Pseudohaemophilus* Segui *et al*. 2026, 15^3^	gen. nov.	*Pseudohaemophilus parahaemolyticus* ^51^	6	[73]
*Pseudohaemophilus minor* (Møller *et al*. 1996) Segui *et al*. 2026, 16^3,58^	comb. nov. [basonym: *Actinobacillus minor* Møller *et al*. 1996]	NM 305 (=CCUG 38923=CIP 105314)	6	[73]
*Pseudohaemophilus parahaemolyticus* (Pittman 1953) Segui *et al*. 2026, 15^3^	comb. nov. [basonym: *Haemophilus parahaemolyticus* Pittman 1953 (Approved Lists 1980)]^34^	ATCC 10014 (=CCUG 3716=DSM 21417)^59^	6	[73]
*Pseudohaemophilus paraphrohaemolyticus* (Zinnemann *et al*. 1971) Segui *et al*. 2026, 16^3^	comb. nov. [basonym: *Haemophilus paraphrohaemolyticus* Zinnemann *et al*. 1971 (Approved Lists 1980)]^34^	ATCC 29237 (=CCUG 3718=DSM 21451)^60^	6	[73]
*Pseudohaemophilus sputorum* (Nørskov-Lauritsen *et al*. 2012) Segui *et al*. 2026, 16^3^	comb. nov. [basonym: *Haemophilus sputorum* Nørskov-Lauritsen *et al*. 2012]^34^	CCUG 13788 (=DSM 24472)	6	[73]
*Pseudomonas artemisiae* Kämpfer *et al*. 2026, 13^3^	sp. nov.	DT-100 (=DSM 115114=LMG 32880)^61^	34	[79]
*Pseudomonas corni* Kämpfer *et al*. 2026, 11^3^	sp. nov.	AK-10 (=CCM 9599=LMG 34325)	34	[79]
*Pseudomonas imperatae* Kämpfer *et al*. 2026, 11^3^	sp. nov.	ST-212 (=CCM 9594=LMG 34330)	34	[79]
*Pseudomonas oplopanacis* Kämpfer *et al*. 2026, 13^3^	sp. nov.	AK-188 (= CCM 9593=LMG 34326)	34	[79]
*Pseudomonas rhizozeae* Kocsubé *et al*. 2026, 5^3^	sp. nov.	SZMC 28357 (=DSM 117247=NCAIM B.02685=NCCB 100969)^62^	56	[80]
*Pseudomonas rosaeacicularis* Kämpfer *et al*. 2026, 11^3^	sp. nov.	AK-381 (=LMG 34445=CCM 9596)	34	[79]
*Pseudomonas salicis* Kämpfer *et al*. 2026, 11^3^	sp. nov.	AK-309 (=CCM 9595=LMG 34328)	34	[79]
*Psychrobacter noctis* Williams *et al*. 2026, 14^3^	sp. nov.	28M-43 (=DSM 112058=NCIMB 15404)	2	[10]
*Rania* Staack *et al*. 2026, 9^3^	gen. nov.	*Rania fehmarnensis*	111	[64]
*Rania fehmarnensis* Staack *et al*. 2026, 10^3^	sp. nov.	SH573 (=CECT 31080=DSM 116763=KCTC 102135)	111	[64]
*Rathayibacter soli* Lee *et al*. 2024, 1480	sp. nov.	SW19 (=KCTC 49860=NBRC 116108)	69	[81]
*Rhizobium flavescens* Su *et al*. 2021, 2171	sp. nov.	FML-4 (=CCTCCAB 2019354=KCTC 62839)	106	[82]
*Rhodocytophaga rosea* Park *et al*. 2020, 2184	sp. nov.	172606-1 (=KCTC 62096=NBRC 114410)	18	[9]
*Roseicella aquatilis* Zhang *et al*. 2022, 4^3^	sp. nov.	NE82 (=KCTC 62412=MCCC 1H00292)	67	[83]
*Roseobacter phycocola* Choi *et al*. 2026, 1^3^	sp. nov.	S98 (=JCM 36648=KACC 23419)	57	[84]
*Roseovarius lacus* Pan *et al*. 2017, 234	sp. nov.	GSS12 (=KCTC 52185=MCCC 1K02302)	104	[85]
*Roseovarius ponticola* Park *et al*. 2019, 220	sp. nov.	W-SW3 (=KCTC 62424=NBRC 113186)^63^	82	[86]
*Saxibacter* Tian *et al*. 2024, 28^64^	gen. nov.	*Saxibacter everestensis*	48	[87]
*Saxibacter everestensis* Tian *et al*. 2024, 282	sp. nov.	ZFBP1038 (=GDMCC 1.3024=JCM 35335)^65^	48	[87]
*Sediminicoccus* Qu *et al*. 2013, 465	gen. nov.	*Sediminicoccus roseus* corrig.	26	[88]
*Sediminicoccus roseus* corrig. Qu *et al*. 2013, 466^66^	sp. nov.	R-30 (=DSM 117172=NBRC 109675)^67^	26	[88]
*Sellimonas caecigalli* corrig. Wongkuna *et al*. 2020, 5^3,68^	sp. nov.	SW451 (=CCOS 1879=DSM 109473)	86	[89]
*Shewanella shenzhenensis* Zhang *et al*. 2022, 1250	sp. nov.	A25 (=GDMCC 1.2731=JCM 34900)	73	[90]
*Shimia fortis* Dong *et al*. 2026, 8^3^	sp. nov.	SDUM112013 (=KCTC 8307=MCCC 1H01481)	1	[91]
*Singulisphaera chitinilytica* Dedysh *et al*. 2026, 1^3^	sp. nov.	PoT (=KCTC 102290=VKM B-3855)	78	[92]
*Singulisphaera photorubra* Dedysh *et al*. 2026, 1^3^	sp. nov.	Ch08 (=LMG 34153=KCTC 102486)	78	[92]
*Sphingomonas flavescens* Lee *et al*. 2024, 4^3^	sp. nov.	PT-12 (=KCTC 92114=NBRC 115717)	10	[93]
*Sphingomonas fuzhouensis* Yao *et al*. 2025, 9^3^	sp. nov.	SGZ-02 (=GDMCC 1.4033=JCM 36769)	85	[94]
*Sphingomonas qomolangmaensis* Liu *et al*. 2022, 21^3,69^	sp. nov.	S5-59 (=GDMCC 1.3193=JCM 35564)	5	[95]
*Spiribacter salilacus* Zhang *et al*. 2023, 6^3^	sp. nov.	C176 (=KCTC 72692=MCCC 1H00417)	58	[96]
*Spirosoma areae* Jang *et al*. 2017, 1150	sp. nov.	Arct 1-12 (=JCM 31025=KCTC 42814)	95	[97]
*Stappia quadratibracata* Williams *et al*. 2026, 13^3^	sp. nov.	28M-7 (=DSM 112057=NCIMB 15403)	2	[10]
*Streptococcus nasisuis* Li *et al*. 2026, 7^3^	sp. nov.	ZY1909104 (=CCTCCAB 2023270=NBRC 116581)	21	[98]
*Streptomyces blattellae* Liu *et al*. 2022, 85	sp. nov.	TRM63209 (=CCTCCAA 2018093=LMG 31403)^70^	55	[99]
*Streptomyces colwelliae* Nouioui *et al*. 2025, 8^3^	sp. nov.	Agncl-13 (=CECT 31215=DSM 118684)	3	[100]
*Streptomyces griseiviridis* Law *et al*. 2023, 13^3^	sp. nov.	MUM 136J (=MCCC 1K04199=NBRC 114249)	97	[101]
*Streptomyces lemnae* Boonchu *et al*. 2026, 321	sp. nov.	DW26H14 (=NBRC 116115=TBRC 17042)	38	[102]
*Streptomyces maoxianensis* Guan *et al*. 2015, 1124	sp. nov.	NEAU-Spg16 (=CGMCC 4.7139=DSM 42137)	101	[103]
*Streptomyces odontomachicola* Somphong *et al*. 2026, 9^3^	sp. nov.	ODS28 (=NBRC 116641=TBRC 18345)	24	[66]
*Streptomyces physcomitrii* Zhuang *et al*. 2020, 8^3,71^	sp. nov.	LD120 (=CCTCCAA 2018049=DSM 110638)	89	[104]
*Streptomyces taiwanensis* Chen *et al*. 2026, 8^3,72^	sp. nov.	KHY26 (=BCRC AC10097=NBRC 116718)^73^	36	[105]
*Streptomyces tuberiscabiei* Inthong *et al*. 2026, 14^3^	sp. nov.	WSLK1-3 (=LMG 33893=TBRC 19150)	75	[106]
*Sulfurimonas sediminis* Wang *et al*. 2021, 820	sp. nov.	S2-6 (=KCTC 15854=MCCC 1A14513)	63	[107]
*Targowskia* Segui *et al*. 2026, 17^3^	gen. nov.	*Targowskia paracuniculus* ^51^	6	[73]
*Targowskia paracuniculus* (Targowski and Targowski 1984) Segui *et al*. 2026, 17^3^	comb. nov. [basonym: *Haemophilus paracuniculus* Targowski and Targowski 1984]^34^	ATCC 29986 (=CCUG 43573=DSM 21452)^74^	6	[73]
*Terrisphaera* Yoon *et al*. 2026, 10^3^	gen. nov.	*Terrisphaera buseokensis*	40	[108]
*Terrisphaera buseokensis* Yoon *et al*. 2026, 10^3^	sp. nov.	23ND18S-11 (=FBCC-B19162=KCCM 90593=NBRC 117123)	40	[108]
*Trinickia camelliae* Wang *et al*. 2026, 6^3^	sp. nov.	YCB016 (=GDMCC 1.3849=JCM 36232)	53	[109]
*Ureibacillus antri* (Narsing Rao *et al*. 2020) Yadav *et al*. 2024, 15^3^	comb. nov. [basonym: *Lysinibacillus antri* Narsing Rao *et al*. 2020]^75^	SYSU K30002 (=CGMCC 1.13504=KCTC 33955)	79	[110]
*Vagococcus zengguangii* Ge *et al*. 2021, 7	sp. nov.	MN-17 (=GDMCC 1.1589=JCM 33478)^76^	91	[111]
*Verrucosispora rhizosphaerae* Xie *et al*. 2018, 131	sp. nov.	2603PH03 (=CCTCCAA 2016023=DSM 45673)	102	[112]
*Vitreimonas silvestris* (Vieira *et al*. 2020) Hallgren *et al*. 2026, 1^3^	comb. nov. [basonym: *Terricaulis silvestris* Vieira *et al*. 2020]	0127_4 (=CECT 9243=DSM 104635)	27	[9]
*Vreelandella halophytica* Romano-Rodríguez *et al*. 2026, 11^3^	sp. nov.	EE7 (=CECT 31281=LMG 34082)	54	[45]
*Vreelandella mediterranea* Romano-Rodríguez *et al*. 2026, 11^3^	sp. nov.	TE19 (=CECT 31275=LMG 34086)	54	[45]
*Vreelandella xerophytica* Romano-Rodríguez *et al*. 2026, 11^3^	sp. nov.	EE22 (=CECT 31280=LMG 34087)	54	[45]
*Zestomonas ipomoeae* Kämpfer *et al*. 2026, 13^3^	sp. nov.	ST-55 (=DSM 115239=LMG 32881)^77^	34	[79]

For references to Validation Lists 1–71, see *Int J Syst Bacteriol* **49** (1999) 1325. Lists 72–197 were published in *Int J Syst Evol Microbiol* **50** (2000) 3, 423, 949, 1415, 1699, 1953; and **51** (2001) 1, 263, 793, 1229, 1619, 1945; and **52** (2002) 3, 685, 1075, 1437, 1915; and **53** (2003) 1, 373, 627, 935, 1219, 1701; and **54** (2004) 1, 307, 631, 1005, 1425, 1909; and **55** (2005) 1, 547, 983, 1395, 1743, 2235; and **56** (2006) 1, 499, 925, 1459, 2025, 2507; and **57** (2007) 1, 433, 893, 1371, 1933, 2449; and **58** (2008) 1, 529, 1057, 1511, 1993, 2471; and **59** (2009) 1, 451, 923, 1555, 2129, 2647; and **60** (2010) 1, 469, 1009, 1477, 1985, 2509; **61** (2011) 1, 475,1011, 1499, 2025, 2563; and **62** (2012) 1, 473, 1017, 1443, 2045, 2549; and **63** (2013) 1, 797, 1577, 2365, 3131, 3931; and **64** (2014) 1, 693, 1455, 2184, 3603; and **65** (2015), 1, 741, 1112, 2017, 2777, 3767; and **66** (2016) 1, 1603, 1913, 2463, 3761, 4299; and **67** (2017) 1, 529, 1095, 2075, 3140, 4291; and **68** (2018), 1, 693, 1411, 2130, 2707, 3379; and **69** (2019), 5, 597, 1247, 1844, 2627, 3313, and **70** (2020) 1, 1443, 2960, 4043, 4844, 5596. Lists 197-230 were published in *Int J Syst Evol Microbiol* **71** (2021) 004600, 004688, 004773, 004846, 004943, 005096; and **72** (2022) 005167, 005260, 005331, 005422, 005517, 005592; and **73** (2023) 005709, 005812, 005845, 005931, 005997, 006080; and **74** (2024) 006173, 006229, 006275, 006398, 006452, 006501; and **75** (2025) 006562, 006640, 006699, 006863, 006910, 006989; and **76** (2026) 007052, 007141, 007224.

1Abbreviations of culture collections cited in this list can be found at https://lpsn.dsmz.de/text/collections.

2Priority number assigned according to the date the documentation and request for validation are received.

3The journal in which the name was effective published does not have continuous pages.

4The list editors have corrected the epithet to *colocynthidis* (co.lo.cyn’thi.dis. L. gen. n. *colocynthidis*, of the colocynth, referring to *Citrillus colocynthis*, …).

5The etymology must state N.L. masc. adj. instead of M.L. neut. adj.

6The list editors have corrected the epithet to *semiaridus* (se.mi.a’ri.dus. L. masc. n. *semis*, the half of anything; L. masc. adj. *aridus*, dry; N.L. masc. adj. *semiaridus*, semi-arid, referring to the environment from which the organism was isolated).

7The list editors have corrected *endophylla* to *endophyllus* (en.do.phyl’lus. Gr. pref. *endo*-, inside; Gr. neut. n. *phyllon*, leaf; N.L. masc. adj. *endophyllus*, living within leaves).

8The protologue heading erroneously states *Acinetobacter *strain sp. nov.

9Preferred etymology: Gr. fem. n. *kystis*, a bladder; N.L. gen. n. *cystesis*, of a bladder.

10Preferred etymology: Gr. neut. n. *ourema*, urine; N.L. gen. n. *urematis*, …

11Preferred syllabification: cre.pus’cu.li.

12Preferred syllabification and etymology: fer.ro.o’xi.dans. L. neut. n. *ferrum*, iron; … N.L. masc. part. adj. *ferrooxidans*, …

13Preferred etymology: … *salinus*, saline; … *hypersalina*, hypersaline.

14The type strain designation LNSP4103-1 is not found in the protologue but elsewhere in the paper. The manuscript has TDS-413 instead of TSD-413.

15 The list editors have corrected *brookingsii *to *brookingsensis *(broo.kings.en’sis. N.L. fem. adj. *brookingsensis*, pertaining to Brookings, South Dakota).

16Preferred etymology: Gr. masc. n. *hals*, salt; …

17The protologue gives B-3564 instead of VKM B-3564.

18The authors gave DSM 13,228 and JCM 12,297 instead of DSM 13228 and JCM 12297.

19The authors gave DSM 23,484 and NBRC 106,234 instead of DSM DSM 23,484 and NBRC 106,234.

20Preferred syllabification and etymology: Ha.lo’co.la. Gr. fem. n. *hals*, the sea; L. masc./fem. suff. -*cola*, inhabitant, dweller; N.L. fem. n. *Halocola*, inhabitant of the sea.

21Preferred etymology: N.L. gen. n. *ammonii*, of ammonium.

22Preferred etymology: Gr. neut. n. *hydor*, water; N.L. pref. *sulfo-*, pertaining to sulfur; N.L. masc. n. *vibrio*, that which vibrates, and also a bacterial genus name; N.L. masc. n. *Hydrosulfovibrio*, a vibrio-shaped bacterium that can disproportionate sulfur and utilize hydrogen as electron source.

23Not *Hydrosulfovibrio ferrireducens *strain CB1MN as given in the protologue.

24Preferred syllabification and etymology: fer.ri.re.du’cens. L. neut. n. *ferrum*, iron; L. pres. part. *reducens*, bringing back, leading back, N.L. masc. part. adj. *ferrireducens*, iron-reducing.

25The protologue heading must state *Janthinobacterium *instead of *J*.

26The authors gave DSMZ instead of DSM.

27The effective publication states that the type strain was also deposited as CCTCC AA 2024017, but no documentation was supplied.

28The effective publication states that the type strain was also deposited as CCTCC AA 2024021 and CGMCC 1.61938, but no documentation was supplied.

29The effective publication states that the type strain was also deposited as CCTCC AA 2024016, but no documentation was supplied.

30The effective publication states that the type strain was also deposited as CCTCC AA 2024015, but no documentation was supplied.

31The effective publication states that the type strain was also deposited as MCCC 1K09192, but no documentation was supplied.

32The effective publication states that the type strain was also deposited as CGMCC 1.61923, but no documentation was supplied.

33The list editors have adopted the spelling correction of *londoniensis *to *londonensis *as given in the protologue heading for the effective publication (lon.don.en’sis. N.L. fem. adj. *londonensis*, pertaining to London, Ontario, Canada, …).

34The basonym was assigned to Risk Group 2 [German TRBA 466, 'Einstufung von Prokaryonten (Bacteria und Archaea) in Risikogruppen'].

35The effective publication states that the type strain was also deposited as ATCC 49954, CCM 4698, CIP 103466, CLIP 12229, JCM 32891 and NCTC 12701, but no documentation was supplied.

36Preferred syllabification and etymology: Mas.si.li.bac.te’ri.um. L. fem. n. *Massilia*, the Roman name of Marseille; N.L. neut. n. *bacterium*, a rod; N.L. neut. n. *Massilibacterium*, a rod from Marseille.

37The authors erroneously gave *Massilibacterium senegalense *strain mt8T as the type species.

38Preferred syllabification and etymology: se.ne.gal.en’se. N.L. neut. adj. *senegalense*, pertaining to Senegal …).

39The list editors have corrected *Massilibacteroïdes *to *Massilibacteroides* with syllabification and etymology: Mas.si.li.bac.te.ro.i’des. L. fem. n. *Massilia*, the Roman name of Marseille; N.L. masc. n. *Bacteroides*, a bacterial genus name; N.L. masc. n. *Massilibacteroides*, a bacterium from Marseille related to *Bacteroides*.

40Preferred syllabification and etymology: bo’vis. L. gen. n. *bovis*, of a cow.

41The protologue gives B-3409 instead of VKM B-3409.

42Preferred syllabification and etymology: L. gen. n. *horti*, of a garden.

43The effective publication erroneously states KCTC 52187, which is *Limibaculum halophilum*.

44The protologue heading states *Luteus* instead of *luteus*.

45The authors gave KCTC 92,119 instead of KCTC 92119.

46The list editors have corrected *aquaegermanicae *to *aquigermanicae *(a.qui.ger.ma’ni.cae. …).

47The etymology must state L. fem. adj. instead of L. fem. Adj.

48The list editors have corrected *bryophyllum *to *bryophylli *(bry.o.phyl’li. N.L. gen. n. *bryophylli*, of *Bryophyllum* …).

49The list editors have corrected the epithet to *rubeinfantis *(ru.be.in.fan’tis. L. masc. adj. *rubeus*, red; L. masc./fem. n. *infans*, infant; N.L. gen. n. *rubeinfantis*, of a red infant, referring to the hair decoloration observed in children with kwashiorkor.

50The effective publication states that the type strain was also deposited as ACAM 630, but no documentation was supplied.

51The information given on the type strain and accession numbers for its gene and genome sequences must be deleted from the protologue as a genus does not have a type strain.

52The effective publication states that the type strain was also deposited as CIP 102513 and NCTC 7857, but no documentation was supplied.

53The effective publication states that the type strain was also deposited as DSM 21203, but no documentation was supplied.

54The effective publication states that the type strain was also deposited as ATCC 49733 and CIP 103890, but no documentation was supplied.

55The list editors have corrected *puniceum *to *puniceus *(pu.ni’ce.us. L. masc. adj. *puniceus*, …).

56Preferred etymology: N.L. neut. n. *pectinum*, …; N.L. fem. adj. *pectinovora*, …

57The effective publication states that the type strain was also deposited as CIP 54.2 and DSM 8925, but no documentation was supplied.

58Preferred etymology: L. comp. masc. adj. *minor* …

59The effective publication states that the type strain was also deposited as CIP 56.86 and NCTC 8479, but no documentation was supplied.

60The effective publication states that the type strain was also deposited as CIP 102512 and NCTC 10670, but no documentation was supplied.

61The effective publication states that the type strain was also deposited as CCM 9281, but no documentation was supplied.

62The NCCB culture collection document has strain Ps_91 instead of SZMC 28357.

63The effective publication states that the type strain was also deposited as KACC 19615, but no documentation was supplied.

64Preferred etymology: L. neut. n. *saxum*, rock; N.L. masc. n. *bacter*, a rod; N.L. masc. n. *Saxibacter*, …).

65The effective publication states that the type strain was also deposited as EE 014, but no documentation was supplied.

66The list editors have corrected *rosea* to *roseus* (ro’se.us. L. masc. adj. *roseus*, rose-coloured).

67The effective publication states that the type strain was also deposited as CGMCC 1.12302, but no documentation was supplied.

68The list editors have corrected the epithet to *caecigalli *(cae.ci.gal’li. L. neut. n. *caecum*, caecum; L. masc. n. *gallus*, a cock; N.L. gen. n. *gallicaeci*, of the caecum of a chicken).

69The etymology must state N.L. fem. adj. instead of N.L. masc. adj.

70The authors gave LMG 31,403 instead of LMG 31403.

71The protologue heading must state *Streptomyces *instead of *S.*

72Preferred syllabification and etymology: tai.wan.en’sis. N.L. masc. adj. *taiwanensis*, …

73The culture collection numbers were not given in protologue but are found elsewhere in the effective publication.

74The effective publication states that the type strain was also deposited as CIP 107045, but no documentation was supplied.

75The title and the protologue heading must state comb. nov. instead of comb. Nov.

76The effective publication states that the type strain was also deposited as CGMCC 1.16726, but no documentation was supplied.

77The effective publication states that the type strain was also deposited as CCM 9283, but no documentation was supplied.

## Corrigenda

Validation list no. 225 [113] erroneously gave DSM 25018 as deposit of the type strain of *Paenirhizobium selenitireducens* (Hunter *et al*. 2008) Ma *et al*. 2023. It should be DSM 25108.

We thank K. Huber-Fischer (Leibniz Institute DSMZ, Braunschweig) for alerting us to this error.

Validation list no. 229 [114] gave Hó5 as original designator of the type strain of *Thiobacillus cuprinus* Huber and Stetter 1990. It should be Hö5.

## Abbreviations

ICNP: International Code of Nomenclature of Prokaryotes
IJSEM: InternationalJournal of Systematic and Evolutionary Microbiology
